# Nasal therapy—The missing link in optimising strategies to improve prevention and treatment of COVID-19

**DOI:** 10.1371/journal.ppat.1010079

**Published:** 2021-11-24

**Authors:** Norman A. Ratcliffe, Helena C. Castro, Izabel C. Paixão, Victor G. O. Evangelho, Patricia Azambuja, Cicero B. Mello

**Affiliations:** 1 Programa de Pós-Graduação em Ciências e Biotecnologia, IB, Universidade Federal Fluminense, Niterói, Brazil; 2 Department of Biosciences, Swansea University, Swansea, United Kingdom; 3 Laboratório de Virologia Molecular e Biotecnologia Marinha, IB, Universidade Federal Fluminense, Niterói, Brazil; 4 Laboratório de Bioquimica e Biotecnologia, IB, Universidade Federal Fluminense, Niterói, Brazil; Boston Children’s Hospital, UNITED STATES

## The bottom line

Recent reports of the transmission of Severe Acute Respiratory Syndrome Coronavirus 2 (SARS-CoV-2) by fully vaccinated people [1] do not undermine the value of injected vaccines that continue to protect against serious illness and hospitalisation. They are, however, an early warning for immediate action to develop new drugs and approaches against Coronavirus Disease 2019 (COVID-19). The logical answer is to target the initial nasal portal of COVID-19 entry into the body with prophylactic drugs, which, together with injected vaccines, could potentially completely prevent infection and subsequent transmission of a range of variants. This paper outlines published work in this vital area in the hope that it becomes an urgent priority for development.

## COVID-19 variants

Over 12,000 mutations have been catalogued in SARS-CoV-2 genomes [2] and have resulted in new SARS-CoV-2 variants, including those identified in South Africa (B.1.351), United Kingdom (B.1.1.7), California (B.1.427 and B.1.429), Brazil (P.1 and P.2), India (B1.617.2 = Delta), Peru (C.37 = Lambda), and Colombia (Mu). Such variants may have increased transmissibility and pathogenicity, higher viral loads, and vaccine resistance [3–5].

## A missed opportunity

Vaccines provide short-term relief from COVID-19, but rapid evolution of resistant viral variants necessitates additional supportive strategies, including broad-spectrum antiviral agents coupled with innovative prophylactic and therapeutic processes. Antiviral agents against SARS-CoV-2 should have been repurposed drugs, but of all the drugs tested, those effective in the later stages of infection, such as dexamethasone, are the main ones granted approval for emergency use [6]. One exception has been monoclonal antibody therapy [7]. An important missing link has been the lack of innovative drug development for treating the early stages of COVID-19 infection. Disease pathology extols studying the initial interactions of invading pathogens with the body, involving adsorption, colonisation, penetration, multiplication, and host innate immunity [8].

## COVID-19 entry portal

The main entry of SARS-CoV-2 occurs in the ciliated epithelium lining the nose [9–11]. The importance of the nasal epithelium in host invasion, involving the specific attachment of influenza and other viruses to the ciliated cells, was reported over 50 years ago [12]. These ciliated cells have the highest expression levels, in the airways of the body, of the SARS-CoV-2 entry receptors, angiotensin converting enzyme 2 (ACE2), and the viral entry-associated protease, transmembrane serine protease 2 (TMPRSS2) [9–11]. SARS-CoV-2 binds to these using the receptor-binding domain (RBD) of the virus spike protein [13]. Following attachment and entry into the nasal epithelium, the virus multiplies, spreading around the body [11]. To emphasise, the ciliated cells of the nasal mucosa are the main host entry targets for the virus, so that denying access of SARS-CoV-2 to the entry receptors by intranasal drug prophylaxis needs prioritising.

## New opportunities—Nasal therapy

Most SARS-CoV-2 vaccines are injected and mainly induce serum immunoglobulin G1 (IgG1), which enters and protects the lungs, leaving the nasal epithelia and upper respiratory tract largely unprotected. Any serum immunoglobulin A1 (IgA1) produced by vaccination is not effectively transported to the secretions of the upper respiratory tract including those of the nasal mucosa [14]. The dynamics of the mucosal immune response to COVID-19 is largely neglected, although the IgA secreted is 7 times more potent than IgG at neutralising SARS-CoV-2 [13–15]. Only natural infections induce both IgG1to protect the lungs as well as IgA1 to protect the upper respiratory tract, including the nasal passages [16]. Thus, injected vaccines fail to fully address the main portal of virus entry into the body through the nose, and, yet, few, if any, drugs have been developed to kill the virus in this early stage.

The nose is therefore likely to remain a source of infective virus transmission even after parenteral vaccination, which fails to completely eliminate the virus in the nose [1,17]. A single intranasal vaccination in rhesus macaques prevented SARS-CoV-2 infection in both the upper and lower respiratory tracts [18]. Parenteral vaccination and nasal therapy combined could realise the ultimate goal of completely eliminating these viral pathogens and sterilising the nose.

## Intranasal drug candidates

Drugs for nasal pharmacological prophylaxis against COVID-19 are under development and include (1) those blocking virus attachment to the host entry receptors without involving host immunity; and (2) intranasal vaccines or immune stimulants eliciting antiviral antibodies and memory cells at the mucosal surface.

- **Category 1:** Include povidone-iodine [19], nitric oxide [20], ethyl lauroyl arginate hydrochloride [21], astodrimer sodium (SPL7013) [22,23], iota-carrageenan [24–26], and many others. These utilise nasal sprays and are at different stages of development globally. One very significant study for prevention of the early phase of SARS-CoV-2 entry into the body utilises poly(lactic-*co*-glycolic acid) nanoparticles to deliver and confine drugs specifically to treat the nasal sinuses with slow release over one week [27]. Stringent published clinical trials of these drugs are needed to satisfy the regulatory bodies as these may become available for sale to the public. Once approved, however, they could have enormous impacts on COVID-19 prophylaxis and therapy, particularly in deprived countries, as they are cheap and convenient and could also deal with breakthrough virus to sterilise the nose. They might be more acceptable too to those refusing injected vaccines.
- **Category 2:** Intranasal vaccines are also being developed, inducing IgA since dimeric forms of these antibodies are particularly potent and found at the mucosal surfaces where SARS-CoV-2 targets the cells [14].

Previous studies to develop nasal therapy for respiratory viruses have met with variable success. For example, a live attenuated flu nasal spray vaccine, called Flu Mist, has been approved by the US Food and Drug Administration (FDA), although the results of clinical trials have been discordant [28]. Developing nasal sprays with some respiratory viruses can be problematic, epitomised by the common cold and the work of David Tyrell [29] who showed that more than 100 different viruses may be involved. SARS-CoV-2, however, is more promising since few variants dominate the pandemic and parenteral vaccines have already been produced. Preclinical and clinical trials with a variety of drugs for nasal therapy against COVID-19 are also underway. For example, the nasal delivery of IgG monoclonal antibodies against SARS-CoV-2 engineered into immunoglobulin M (IgM) antibodies protect against virus variants in rats [30], while intranasal vaccination with the AstraZeneca vaccine, AZD1222, reduces virus concentrations in nasal swabs in 2 different SARS-CoV-2 animal models [31]. Furthermore, transgenic mice receiving one intranasal dose of an adenovirus-vectored vaccine, ChAd-SARS-CoV-2-S, also conferred superior immunity to SARS-CoV-2 than 2 intramuscular injections and evidenced sterilisation immunity in the upper respiratory tract [32]. Additional progress has been made in India with the approval of a human Phase II clinical trial of a COVID-19 nasal vaccine [33]. There will inevitably be delays and setbacks due to our lack of understanding of the dynamics of intranasal vaccination for COVID-19 so that additional research is urgently required [14,34,35]. Meanwhile, some Category 1 drugs may be approved more rapidly and available to prevent viral shedding following full vaccination against Delta and other variants [23–25].

In conclusion, nasal therapy has great potential to prevent and treat a variety of respiratory viruses. As patients present at different stages of COVID-19 or with other viral infections, we will need a selection of therapeutic strategies from vaccines to broad-spectrum antiviral drugs, delivered in different ways from injection, sprays/inhalations, and tablets alone or in combinations, to counter these threats.
